# Obstructive Sleep Apnoea Modulates Airway Inflammation and Remodelling in Severe Asthma

**DOI:** 10.1371/journal.pone.0150042

**Published:** 2016-03-02

**Authors:** Camille Taillé, Anny Rouvel-Tallec, Maria Stoica, Claire Danel, Monique Dehoux, Viviana Marin-Esteban, Marina Pretolani, Michel Aubier, Marie-Pia d’Ortho

**Affiliations:** 1 Service de Pneumologie A et Centre de Compétence des Maladies Pulmonaires Rares, Hôpital Bichat, Assistance Publique-Hôpitaux de Paris, Paris, France; 2 Centre du Sommeil, Service de Physiologie–Explorations Fonctionnelles, Hôpital Bichat, Assistance Publique-Hôpitaux de Paris, Paris, France; 3 Laboratoire d’Anatomie et Cytologie Pathologiques Hôpital Bichat, Assistance Publique-Hôpitaux de Paris, Paris, France; 4 Laboratoire de Biochimie, Hôpital Bichat-Claude Bernard Hôpital Bichat, Assistance Publique-Hôpitaux de Paris, Paris, France; 5 Faculté de Médecine, Université Paris Diderot, Paris, France; 6 Inserm UMR 1152, Paris, France; 7 Département Hospitalo-Universitaire *FIRE*, Paris, France; 8 Laboratoire d’Excellence *INFLAMEX*, Paris, France; 9 Inserm UMR-S 996, Faculté de Pharmacie, Université Paris sud, Châtenay-Malabry, France; University of Athens, GREECE

## Abstract

**Background:**

Obstructive sleep apnoea (OSA) is frequently observed in severe asthma but the causal link between the 2 diseases remains hypothetical. The role of OSA-related systemic and airway neutrophilic inflammation in asthma bronchial inflammation or remodelling has been rarely investigated. The aim of this study was to compare hallmarks of inflammation in induced sputum and features of airway remodelling in bronchial biopsies from adult patients with severe asthma with and without OSA.

**Materials and Methods:**

An overnight polygraphy was performed in 55 patients referred for difficult-to-treat asthma, who complained of nocturnal respiratory symptoms, poor sleep quality or fatigue. We compared sputum analysis, reticular basement membrane (RBM) thickness, smooth muscle area, vascular density and inflammatory cell infiltration in bronchial biopsies.

**Results:**

In total, 27/55 patients (49%) had OSA diagnosed by overnight polygraphy. Despite a moderate increase in apnoea-hypopnoea index (AHI; 14.2±1.6 event/h [5–35]), the proportion of sputum neutrophils was higher and that of macrophages lower in OSA than non-OSA patients, with higher levels of interleukin 8 and matrix metalloproteinase 9. The RBM was significantly thinner in OSA than non-OSA patients (5.8±0.4 vs. 7.8±0.4 μm, p<0.05). RBM thickness and OSA severity assessed by the AHI were negatively correlated (rho = -0.65, p<0.05). OSA and non-OSA patients did not differ in age, sex, BMI, lung function, asthma control findings or treatment.

**Conclusion:**

Mild OSA in patients with severe asthma is associated with increased proportion of neutrophils in sputum and changes in airway remodelling.

## Background

Severe asthma is a morbid condition, affecting about 3.6% to 10% of people with asthma. The probability of OSA is high for 26% of patients with severe asthma [1]. When evaluated by overnight polysomnography, OSA prevalence (defined by apnoea-hypopnoea index [AHI] ≥15 events/h) is 88% in patients with severe asthma and 58% in those with moderate asthma [2]. Obstructive sleep apnoea (OSA) is considered to contribute to asthma exacerbation [1], daytime and night-time asthma symptoms [3, 4] and poor quality of life [5]. The relationship may be bidirectional, because asthma may also affect the occurrence of OSA [6].

Despite the well-described association between asthma and OSA, the mechanisms that link the 2 diseases remain hypothetical. Obesity or gastroesophageal reflux disease (GERD) may have a role, with reciprocal interactions. Recently, the role of OSA-associated changes in airway inflammation and remodelling has been suggested: in ovalbumin-sensitized rats, chronic intermittent hypoxia modulated both inflammation (by decreasing eosinophil proportion) and remodelling in distal airways [7]. In humans, OSA is associated with increased prevalence of neutrophilic pattern in the sputum [1]. These data suggest that bronchial and/or systemic inflammation related to OSA or intermittent hypoxia may affect bronchial inflammation and remodelling in asthma involving neutrophils, as was described in the cardiovascular system [8] and in upper airways [9].

The aim of this study was to compare features of airway inflammation and remodelling in both induced sputum and bronchial biopsies from patients with severe asthma with and without OSA.

## Materials and Methods

### 1. Patients

The study was prospectively conducted in adult patients referred to our Severe Asthma Clinic who were consecutively recruited during a 12-month period. The asthma diagnosis was confirmed by 2 pulmonary physicians trained in difficult-to-treat asthma. All patients with severe uncontrolled asthma according to Global Initiative for Asthma (GINA) guidelines and who complained of nocturnal respiratory symptoms (snoring, nocturnal asthma), poor sleep quality and/or diurnal symptoms (fatigue or sleepiness) were enrolled in a sleep study. Patients receiving continuous positive airway pressure (CPAP) treatment at the time of the initial appointment and smokers or ex-smokers (>10 packs/year) were excluded. Fibre-optic bronchoscopy and induced sputum analysis are routinely performed for patients with severe asthma in our centre and are proposed to all patients investigated for difficult-to-treat asthma.

Asthma control was evaluated by the Asthma Control Test. Atopy was defined as a positive skin prick test result and/or positive for specific IgE in the blood. Nasal polyposis was diagnosed by a laryngologist. Diabetes, depression and hypertension were considered if specific treatment was required. Data from the visit closest to the night recording were used.

All patients gave written informed consent for follow-up in the COBRA cohort (Cohorte Obstruction Bronchique et Asthme) according to the procedure validated by the local Ethics Committee (Comité de Protection des Personnes d’Ile de France), which approved the study. Consent is usually retained in the patient’s medical file.

### 2. Overnight polygraphy

Nocturnal polygraphy was performed with a portable, computerised data-acquisition system (Embletta, ResMed, Saint-Priest, France). The system records oronasal airflow from nasal pressure (nasal flow) and a mouth thermistor (oral flow), chest and abdominal effort by inductance plethysmography, pulse oximetry, snoring, actimetry and body position [10]. Recording was performed in the hospital or at home, depending on the patient preference. In terms of trace quality, we considered only recordings of ≥ 4 h duration, with no missing traces. Scoring was performed manually, according to international recommendations.

Apnoea was defined as a decrease of > 90% in nasal airflow, lasting at least 10 s. Hypopnea was defined by ≥ 30% decrease in nasal flow associated with 4% desaturation (recommended definition) or ≥ 50% decrease associated with 3% desaturation or an arousal (alternate definition), as previously described [11].

The number of apnoea-hypopnoea events per hour (AHI) was determined after the exclusion of periods with movements, considered periods of wakefulness. Sleep-disordered breathing was diagnosed as AHI ≥ 5/h. Nocturnal polygraphy was performed at least 2 weeks after recovery from any asthma exacerbation or intervention. The Epworth score and depression score (Center for Epidemiological Studies-Depression, CES-D) were collected. The 20 items in the CES-D scale measure symptoms of depression in 9 different groups on a scale from “0” (not at all or less than one day) to “3” (nearly every day). A total score ≥ 17 for men and ≥ 24 for women indicates depression symptoms. Fatigue was measured by the Pichot questionnaire [12], consisting of 8 questions on fatigue scored progressively from "0" (not at all) to "4" (extremely). A value ≥ 20 indicates fatigue.

### 3. Fibre-optic bronchoscopy and airway remodelling assessment

Four biopsies were collected for each patient during fibre-optic bronchoscopy as described [13]. Biopsies were fixed in 10% formaldehyde, embedded in paraffin and cut into 5-μm sections. Computer-assisted image analysis was used to assess reticular basement membrane thickness (RBM, in micrometres). In addition, total biopsy and airway smooth muscle (ASM) area (in micrometres squared and percentage of total biopsy area, respectively) and number of vessels/μm^2^ were determined after α-actin and CD31 immunostaining, respectively. Eosinophil cationic protein (eosinophils), elastase (neutrophils), CD3 (lymphocytes) and tryptase (mast cells) were detected in bronchial submucosa by immunohistochemistry with the alkaline phosphatase method, and stained cells were counted were measured in a zone of 60-μm depth along the length of the basement membrane.

Induced sputum was analyzed, and macrophages, eosinophils and neutrophils were counted as described [14]. In addition, we measured interleukin 5 (IL-5), IL-4, IL-8, platelet-derived growth factor-BB (PDGF-BB), vascular endothelial growth factor (VEGF) and matrix metalloproteinase 9 (MMP-9) levels by using a multiplex analyser (Milliplex, Merck Millipore, France), according to the manufacturer’s instructions. Elastase activity was quantified with an enzymatic kinetic method [15].

### 4. Statistical analysis

Data were analysed by using GraphPad Prism 5.01 (GraphPad Software, La Jolla, CA, USA) and are expressed as number (%) or mean±SEM and range. Comparisons between 2 groups involved the Mann–Whitney test and Kruskall–Wallis test for continuous variables. Pearson chi-square test was used for comparing categorical variables. Univariate correlation (Spearman's rank-order method) was used for correlation analysis. *P*≤0.05 was considered statistically significant. Original data are available as an Excel file in S1 Original Data.

## Results

### 1. Patient characteristics

In total, 55 patients consecutively referred for difficult-to-treat asthma and complaining of nocturnal symptoms, underwent overnight polygraphy (Table 1). All had severe uncontrolled asthma despite high doses of inhaled steroids associated with a long-acting beta-agonist. Patients were mainly overweight women with late-onset asthma and minimally impaired lung function. About one third were obese. Almost half had atopic disease. A total of 23% (13/55) required daily oral steroid treatment. The mean Epworth score was 11.4±0.8 and mean Pichot score 20.4±1.2 (Table 2).

**Table 1:** Characteristics of asthma patients with and without obstructive sleep apnoea (OSA).

	All patients (n = 55)	OSA (n = 27)	Non-OSA (n = 28)
**Age (years)**	47.8±1.7 [17–78]	51.2±2.5 [24–78]	44.6±2.2 [17–70]
**Male (n/%)**	12/21.8%	7/26.0%	5/17.8%
**BMI (kg/m**^**2**^**)**	28.4±0.8 [20–50.6]	29.6±1.4 [20.6–50.6]	27.2±0.8 [20–37.4]
**BMI >30 (n/%)**	16/29%	8/29.6%	8/28.5%
**Age at asthma onset (years)**	23.2±4.0 [1–70]	28.4±4.0 [1–70]	17.6±3.0 [1–45] *
**Asthma control test**	10.3±0.8 [6–22]	10.01 ± 0.9 [6–20]	10.5 ± 1.0 [5–22]
**Daily oral steroid use (n)**	13/23.6%	7/26.0%	6/21.4%
**Inhaled steroid dose (μg/d)**	1902±41 [1600–2000]	1927±33 [1600–2000]	1878±74 [1600–2000]
**Antileukotriene agent**	45/82%	23/85%	22/78.5%
**Nasal steroid**	44/80%	18/66.6%	26/92.8%*
**Antihistamine**	33/60%	9/33%	24/85%*
**Atopy (n/%)**	29/52.7%	7/26%	22/78.5% *
**Chronic rhinitis (n/%)**	44/80%	18/66%	26/93%*
**Nasal polyposis (n/%)**	18/32.7%	6/22%	12/43%
**GERD symptoms (n/%)**	40/72.7%	21/78%	19/68%
**Depression (n/%)**^$^	16/29%	9/33%	7/25%
**Hypertension (n/%)**	15/27%	11/41%	4/14%*
**Cerebrovascular disease (n/%)**	4/7%	4/15%	0/0%*
**Diabetes (n/%)**	4/7%	3/11%	1/3.5%
**Dyslipidaemia (n/%)**	3/5%	1/4%	2/7%
**FEV1 (% predicted)**	65.2±2.3 [24–116]	69.9±4.2 [24–118]	67.4±4.5 [28–116]
**FEV1/FVC (%)**	61±2.7 [27–92]	62.1±2.2 [40–84]	61.1±2.7 [27–92]
FEV1 reversibility (n/%)**	21/38%	9/33%	12/43%

Data are mean±SEM and range or no. (%).

* p<0.05 compared with OSA.

** Reversibility observed at the time of evaluation.

^$^ Proportion of patients receiving antidepressant drugs.

**Table 2:** Characteristics of OSA for OSA and non-OSA patients.

	All patients	OSA	Non-OSA
	(n = 55)	(n = 27)	(n = 28)
**Pichot score**	20.4±1.2 [3–32]	22.0±1.5 [3–32]	15.4±1.7 [4–24]*
**Pichot ≥20 (n/%)**	21/38%	14/52%	7/25%*
**Epworth score**	11.4±0.8 [0–24]	10.4±1.0 [0–18]	12.2±1.2 [0–24]
**Epworth ≥11 (n/%)**	22/40%	12/44%	10/36%
**Epworth ≥11 and Pichot ≥20 (n/%)**	10/18%	7/26%	3/10.7%
**Depression scale**	22.9±2 [0–49]	23.2±2.5 [0–49]	22.5±2.1 [8–44]
**AHI (/h)**	9.1±1 [0–35]	14.2±1.6 [5–35]	2.0±0.2 [0–4]*
**AHI ≥ 15/h (n/%)**	9/16%	9/33%	NA
**AHI ≥ 30 (n/%)**	3/5%	3/11%	NA
**Minimal SpO**_**2**_	85.2± 0.9 [65–95]	85.4±0.8 [76–91]	86.3±1.9 [65–95]
**SpO**_**2**_	94±0.2 [90.4–97.8]	93.5±0.2 [90.4–95.3]	95.0±0.4 [92.3–97.8]*
**Index desaturation >3%/h**	7.7±1.2 [0.3–36.9]	12.6±1.6 [1.4–36.9]	2.2±0.5 [0.3–12.1]*
**Proportion of time with SpO**_**2**_ **<90%**	2.5±1 [0–32]	4.0±1.7 [0–32]	0.7±0.3 [0–5.7]*

AHI, apnoea-hypopnoea index

Data are mean±SEM and [range]

* p<0.05 compared with OSA.

### 2. OSA prevalence and characteristics

Nocturnal polygraphy was performed in the hospital for 15 patients (9 non-OSA and 6 OSA) and at home for 40. Patient characteristics did not differ by the location of testing. In total, 27/55 patients (49%) had OSA. Among them, 9 patients (33%) had AHI ≥15/h and 3 had AHI ≥30/h (Table 2). All patients, even those without OSA, showed oxygen desaturation during the night, but, as expected, SpO_2_ value was significantly lower for OSA than non-OSA patients. The Epworth score and depression score did not differ between OSA and non-OSA patients. The proportion of patients with Epworth score ≥11 was similar in the 2 groups. The mean Pichot fatigue score was higher for OSA than non-OSA patients (p = 0.006) and the proportion with a Pichot score ≥20 was higher for OSA patients (p = 0.04).

OSA and non-OSA patients did not differ in age, sex ratio, BMI, lung function, asthma control test or treatments (Table 1), except for age at onset of disease, which was higher in patients with OSA. The frequency of GERD symptoms did not differ between OSA and non-OSA patients (78% and 68%, respectively), and the proportion of anti-reflux therapy was similar in both groups. The proportion of hypertension and cerebrovascular disease was significantly higher for OSA than non-OSA patients. Atopic disease was more frequent for non-OSA than OSA patients (78.5% vs 26%, p<0.001), as was chronic rhinitis (p = 0.005). Antihistamine drug use was more frequent for non-OSA than OSA patients.

### 3. OSA is associated with specific remodelling and airway neutrophilic inflammation

**Fig 1:**
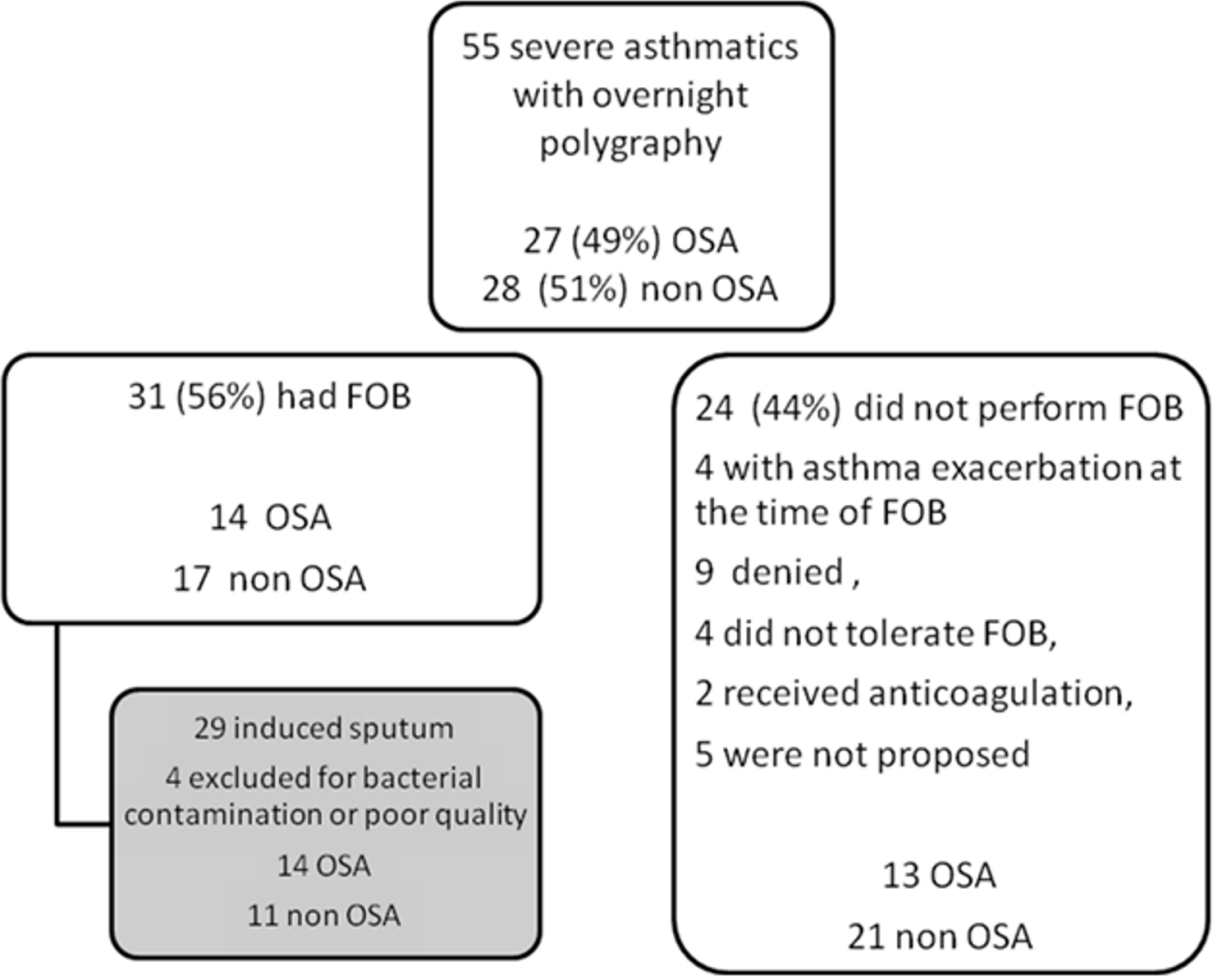
Flow of participants through the study. FOB: fibre optic bronchoscopy.

A total of 31 patients underwent fibre-optic fibroscopy and bronchial biopsies. Reasons for not performing these tests are in Fig 1. These patients did not differ from those who did not undergo endoscopy in terms of age, sex, BMI, asthma-associated diseases or AHI. ASM area and number of vascular sections were similar for OSA and non-OSA patients. The RBM was significantly thinner for OSA than non-OSA patients (Table 3). The number of eosinophils, neutrophils, mast cells and lymphocytes in biopsies was similar between the 2 groups, despite the number of neutrophils being higher, although not significantly, in OSA than non-OSA patients (Table 3). Macrophages were not quantified.

**Table 3:** Airway remodelling and inflammation features in bronchial biopsies.

	OSA (n = 14)	Non-OSA (n = 17)
**Reticular basement membrane thickness (μm)**	5.8±0.4	7.8±0.4*
**Smooth muscle area (%)**	12.6±2.2	12.35±1.9
**Vessels/mm**^**2**^	62.78±12.6	42.49±7.6
**Eosinophils/mm**^**2**^	5.2±3.5	7.8±3.6
**Neutrophils/mm**^**2**^	51.8±17.6	34.8±10.5
**Lymphocytes/mm**^**2**^	64.1±29.4	70.4±14.6
**Mast cells/mm**^**2**^	43.5±14.7	78.5±21.1

Data are mean±SEM.

*p<0.05, compared with OSA.

Among patients who had fibre-optic bronchoscopy, 29 underwent induced sputum analysis (Fig 1). Induced sputum was of poor quality in 4 cases, with squamous cell contamination or evidence for bacterial infection, and was excluded from subsequent analyses. OSA and non-OSA patients had a high but similar proportion of eosinophils and lymphocytes in sputum, but the proportion of neutrophils was higher and that of macrophages lower for OSA than non-OSA patients (Table 4).

**Table 4:** Airway inflammation variables in induced sputum from OSA and non-OSA patients.

	OSA (n = 14)	Non-OSA (n = 11)
**IL-4 level (pg/ml)**	40±19.5	37±.6
**IL-5 level (pg/ml)**	6±1	10.05±1.5*
**IL-8 level (pg/ml)**	13,800±3,366	3,195±923.0*
**VEGF level (pg/ml)**	233.4±42.0	382.9±50.08*
**PDGF level (pg/ml)**	67.14±18.8	113.8±15.55
**Elastase activity (nM Ela)**	13.2±11.1	5.54±2.7
**MMP-9 level (pg/ml)**	88,879±16,292	29,225±11,191*
**Macrophages (%)**	38.7±7.1	59.8±6.5*
**Lymphocytes (%)**	4.9±2.6	5.2 ±1.5
**Eosinophils (%)**	6.5±2	13.4±5.3
**Neutrophils (%)**	50±8.3	20.7±5.8*

Data are mean±SEM.

* P<0.05 compared with OSA.

OSA patients showed a higher proportion of neutrophils along with higher IL-8 level in sputum as compared with non-OSA patients, but IL-5 and VEGF levels were higher in non-OSA than OSA patients (Table 4). As expected sputum neutrophil proportion was correlated with IL-8 level (rho = 0.68, p = 0.0076) but not VEFG or PDGF levels. The proportion of eosinophils was correlated with IL-5 level (rho = 0.67, p = 0.0063).

Elastase activity in sputum was low (under the detection level in many patients) and did not differ between the OSA groups. MMP-9 level in sputum was higher in OSA than non-OSA patients (Table 4) and was correlated only with IL-8 level (rho = 0.8, p<0.001) but not RBM thickness or neutrophils.

**Fig 2:**
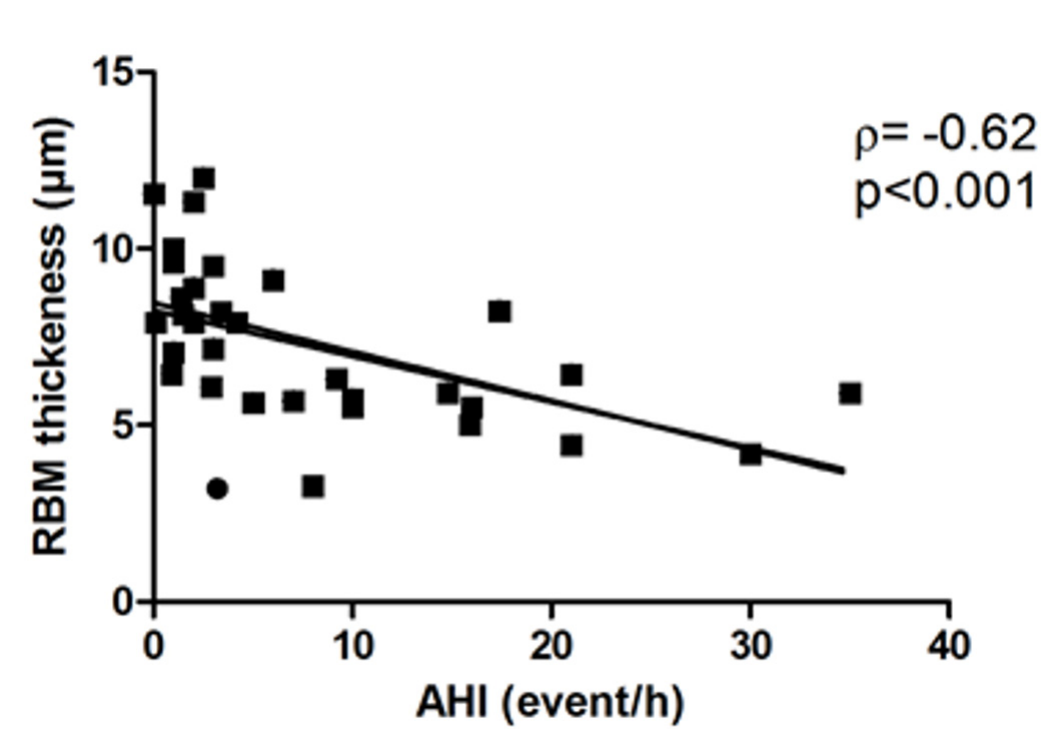
Correlation between reticular basement membrane (RBM) thickness and apnoea-hypopnoea index (AHI).

RBM thickness was not correlated with eosinophil or neutrophil proportion in sputum or biopsies but was negatively correlated with OSA severity, as assessed by AHI (rho = -0.65, p<0.05) (Fig 2). In addition, AHI was correlated with IL-8 level (rho = 0.72, p = 0.006). No other correlation was found between other sleep indices (mean SpO_2_, minimal SpO_2_, desaturation index) and other features of remodelling (vessels, smooth muscle area) or inflammation assessed in sputum or biopsies.

## Discussion

Using induced sputum and bronchial biopsies, we found that OSA in patients with severe asthma is associated with specific features of airway inflammation and remodelling. The disease was characterized by a higher proportion of neutrophils and a lower proportion of macrophages as compared with non-OSA patients, elevated levels of IL-8 and MMP-9 in sputum, and thinner bronchial basement membrane, which was inversely correlated with AHI, a measure of OSA severity. These data suggest that OSA, although mild in most patients with asthma, may modulate airway inflammation and remodelling in the disease.

The hallmark of neutrophilic inflammation in airways of OSA patients was previously reported in induced sputum and exhaled breath condensate [16–18] and associated with elevated IL-8 level [18]. These features have all been associated with OSA severity in previous studies and confirmed in the present work. We recently demonstrated that airway epithelial cells, when exposed *in vitro* to chronic intermittent hypoxia, produce IL-8, contributing to neutrophil recruitment [19]. Of note, delayed apoptosis and increased expression of adhesion molecules have been described in neutrophils from OSA patients [20]. In asthma, increased number of bronchoalveolar lavage or sputum neutrophils is observed only in a subset of patients. This neutrophilic inflammation is associated with asthma severity, increased airflow obstruction and increased rate of asthma exacerbations [21] and contributes to steroid resistance and the pathophysiology of the disease by releasing MMP-9, elastase, leukotriene B4, and platelet-activating factor, which work to enhance the activity of eosinophils [22]. Smokers and ex-smokers were excluded from our study because tobacco exposure can affect airway inflammation, especially increase the number of neutrophils [23]. In asthma, epithelial cells generate pro-neutrophilic factors with chemotactic and apoptosis-delaying actions [24] [25]. Therefore, intermittent hypoxia related to OSA can be another trigger that can induce and/or aggravate neutrophil recruitment into airways in asthma and contribute to increased asthma severity when associated with OSA [1].

OSA-related inflammation can drive tissue remodelling, mainly through increased oxidative stress. In the heart, atrial and ventricular remodelling is observed in OSA patients and can be reversed by continuous positive pressure. Myocyte hypertrophy, fibrosis and increased apoptosis are observed in animal models of chronic intermittent hypoxia [26]. OSA patients can show strongly reduced connective tissue in the upper airways resulting in reduced anchorage between the epithelium and lamina propria [27], but data on lower airway remodelling in OSA are lacking. We observed thinner RBM in patients with asthma and OSA than in non-OSA patients. RBM thickening contributes to airway narrowing and fixed airway obstruction and is associated with steroid resistance [28]. Increased RBM is thought to be secondary to both repeated bronchoconstriction [29] and eosinophilic inflammation [30, 31] because asthma patients without eosinophilic inflammation did not show increased RBM thickness. However, our observations suggest that neutrophils may counteract mechanisms leading to RBM thickening. We found higher MMP-9 level and neutrophil proportion in OSA than non-OSA patients, so the predominant neutrophilic inflammation could contribute to RBM degradation and thinner RBM in this group. However, the neutrophil count in sputum was not correlated with MMP-9 level or RBM thickness. Indeed, neutrophil count in the bronchial mucosa was higher but not significantly in OSA than non-OSA patients. A role of biopsy sampling cannot be excluded to explain why OSA and non-OSA patients differed in neutrophil count in sputum but not on biopsy. However, our hypothesis is supported by recent data in a model of chronic intermittent hypoxia in ovalbumin-sensitized and -challenged rats. After 30 days, increased matrix degradation was observed in distal airways, associated with a change to a predominantly Th1 cellular phenotype [7]. In this model, neutrophil number in bronchoalveolar lavage fluid did not change, and the authors suggested a role of M2 macrophages in matrix deposition. Before definitive conclusions can be drawn about the mechanisms leading to changes in basement membrane thickness, we caution that our study was restricted to proximal airways and did not investigate the distal compartment as in the Broytman et al. study [7].

The last observation in our study that may be related to OSA-associated neutrophilic inflammation is the greater prevalence of hypertension and cerebrovascular diseases in severe asthmatics with OSA without OSA. The causal association between OSA and cardiovascular disease has been linked to oxidative stress and intermittent hypoxia-induced vascular inflammation [32] and characterised by neutrophil activation, decreased neutrophil apoptosis, high IL-8 level, and increased interactions between leukocyte and endothelial cells. This systemic low-grade inflammation may be in addition to the neutrophilic airway inflammation that we found in patients with asthma and OSA, to further increase the cardiovascular risk observed in these patients [33, 34], together with obesity, high dose of oral steroids and limited physical activity. Further studies are required to strengthen this association.

Together, all these findings support that patients with severe asthma with OSA may show a specific phenotype of neutrophilic asthma that needs to be identified to better target treatment. Indeed, increasing steroids doses may have a potential deleterious effect in the setting of neutrophilic /non-eosinophilic inflammation and should be cautioned in this setting. Steroids contribute to neutrophilic airway infiltration by decreasing neutrophil apoptosis, which could contribute to increased OSA risk in this population [35] and to increased OSA severity. OSA, in turn, contributes to the neutrophilic inflammation and therefore to steroid resistance and asthma severity, for a vicious cycle. Some data suggest that continuous positive airway pressure (CPAP) in small series of asthma patients with moderate-to-severe OSA (AHI >15/h) improved asthma symptoms and quality of life [36]. In our series, only 9 patients had OSA with AHI> 15/h, and 3 of these had AHI > 30/h. Whether CPAP could improve asthma control or airway inflammation in patients with severe asthma and mild OSA remains to be investigated.

The association between asthma and OSA is bidirectional, since asthma may contribute to OSA occurrence [6] and the two diseases share common risk factors [37], such as GERD [38] and obesity [39]. In our series, a reciprocal relationship between GERD and OSA may be possible because patients in both groups complained of GERD symptoms with a similar prevalence. Our patients were not morbidly obese and the BMI in each group was similar, despite a higher BMI, although not significant, in patients with OSA than without OSA, which rules out a major role for obesity in the occurrence of OSA in our patients.

The contribution of nasal obstruction in airway obstruction during OSA has been suggested in patients without asthma [40] and with asthma [41]. Surprisingly, we observed a lower prevalence of chronic rhinitis symptoms in OSA than non-OSA patients as well as a lower prevalence of patients receiving a nasal steroid spray and oral antihistamines. Because we did not assess rhinitis score, we cannot rule out that some OSA patients may have had persistent nasal obstruction that contributed to sleep-disordered breathing. The low prevalence of atopic disease in OSA patients is also intriguing. No relationship between OSA and atopy is known, but Tien et al. reported that OSA was associated with an increased and unexplained risk of atopic dermatitis [42]. However, in an animal model of allergic asthma with ovalbumin-sensitized rats, chronic intermittent hypoxia suppressed baseline eosinophil numbers in bronchoalveolar fluid [7].

Our study contains some limitations. First not all severe asthmatics from our clinic were explored but only those who complained of nocturnal symptoms or symptoms usually related to poor sleep (nocturnal asthma symptoms, poor sleep quality, tiredness, sleepiness). OSA was diagnosed in about half of these patients with uncontrolled severe asthma. This rate of OSA is high as compared to the general population, especially considering that most of our patients were female, but the proportion was lower than in a previous study of patients with severe asthma [2, 43, 44]. This discrepancy could result from the use of polygraphy in our study rather than polysomnography. We excluded movement periods corresponding to awake periods from the analysis, which decreased this bias. Another possible explanation for the discrepancy is the higher proportion of women in our study.

To conclude, we demonstrate that OSA is associated with specific changes in airway inflammation associated with predominant neutrophilic inflammation with high levels of MMP-9 and IL-8 and low RBM thickness in patients with severe asthma. Whether CPAP, a specific treatment for this subgroup of patients, could affect these structural changes and therefore the asthma course remains to be investigated.

## Supporting Information

S1 Original Data Orginal data are provided in an Excel data sheet. (XLS)

## Abbreviations

AHI: apnoea-hypopnoea index
CPAP: continuous positive airway pressure
FEV1: forced expiratory volume in one second
OSA: obstructive sleep apnoea
RBM: reticular basement membrane
SDB: sleep-disordered breathing
VEGF: vascular-endothelial growth factor
PDGF-BB: platelet-derived growth factor-BB
MMP-9: matrix metalloproteinase-9
